# Comparison of Fluoroscopy Use Between Two Radiographic Overlay Techniques for Limb Length and Femoral Offset Assessment During Direct Anterior Approach Total Hip Arthroplasty

**DOI:** 10.7759/cureus.116051

**Published:** 2026-09-10

**Authors:** Shivam H Patel, Alexander C Chong, Parth A Vyas

**Affiliations:** 1 Orthopaedic Surgery, Sanford Health, Fargo, USA

**Keywords:** anterior approach, arthroplasty, bmi, hip, radiation exposure

## Abstract

Background: Restoration of leg length and femoral offset is critical to optimizing outcomes following total hip arthroplasty (THA). Several intraoperative techniques are available to assess these parameters when utilizing the direct anterior approach (DAA), many of which rely on fluoroscopy. However, relative radiation exposure associated with these strategies has not been well characterized. This study aims to compare intraoperative radiation exposure between two techniques for limb length and offset assessment during DAA-THA.

Methods: A retrospective chart review was conducted of 159 primary cementless DAA-THA procedures performed by a single fellowship-trained arthroplasty surgeon using either the contralateral or ipsilateral hip for length and offset reference. Medical records were reviewed to record fluoroscopy time, radiation dose, and operative time for each case.

Results: No statistical difference in fluoroscopy time was noted between contralateral (60.4 ± 21.2 seconds) and ipsilateral (55.9 ± 19.6 seconds) techniques (P = 0.17). Similarly, no statistical difference was noted for radiation exposure between contralateral (15.4 ± 13.3 mGy) and ipsilateral (13.0 ± 9.0 mGy) techniques (P = 0.17). However, when adjusted for operative time and presented as arbitrary units (AU), the ipsilateral technique was associated with less fluoroscopy time (0.34 ± 0.1 AU, P = 0.0025) and radiation exposure (0.08 ± 0.05 AU, P = 0.045) when compared to contralateral fluoroscopy time (0.39 ± 0.1 AU) and radiation exposure (0.1 ± 0.07 AU). Stratified analysis by body mass index (BMI) demonstrated a significant main effect of BMI on radiation exposure (P < 0.001) and operative time (P < 0.001). Regardless of technique, increasing BMI was positively correlated with higher radiation dose (r= 0.52, P < 0.001) and longer operative time (r = 0.42, P < 0.001).

Conclusion: Both reference techniques had similar non-adjusted fluoroscopy time and exposure during DAA-THA; however, when normalized by operative time, the ipsilateral technique may require less fluoroscopy. BMI significantly influenced radiation dose and operative duration for both techniques. Ongoing critical evaluation of surgical techniques is essential to optimize safety, particularly when modifications may reduce radiation exposure to patients and operating room personnel.

## Introduction

Total hip arthroplasty (THA) is a highly effective surgical procedure performed to relieve pain, restore mobility, improve hip stability, and enhance overall quality of life in patients with end-stage osteoarthritis, traumatic or pathological fractures, congenital hip disorders, and inflammatory arthritis [1]. Despite its success, postoperative limb length discrepancy remains one of the leading causes of patient dissatisfaction and litigation following THA [2-4]. Although minor limb length discrepancies are not uncommon after THA, significant discrepancies (typically ≥1 cm) have been associated with complications such as gait disturbance, pain, and potential nerve palsy [5,6]. Consequently, accurate determination and correction of limb length while preserving joint stability represent key intraoperative challenges that surgeons must address during THA [1,7].

Several preoperative and intraoperative strategies have been described to evaluate limb length and femoral offset during direct anterior approach total hip arthroplasty (DAA-THA), many of which involve the use of a stable pelvic reference point and a variable femoral reference point [8]. Preoperative templating as a standalone tool is not sufficiently effective for achieving the desired limb length during THA, with some studies suggesting that implanted THA components match the preoperatively templated components in only 60% of cases, although this is largely dependent on the experience and skill of the individual performing the templating [8,9]. While preoperative templating still holds value, such as for anticipating potential intraoperative difficulties and assessing implant system compatibility, most orthopedic surgeons elect to use intraoperative fluoroscopic imaging during DAA-THA with trial implants to assess limb length and femoral offset before placement of the final implants.

The use of fluoroscopy during intraoperative limb length assessment is an important consideration for patients, surgeons, and operating room staff, particularly in the context of cumulative lifetime radiation exposure. Previous studies have demonstrated that orthopedic surgeons who routinely use fluoroscopy may face up to a fivefold increase in lifetime cancer risk [10-12]. Despite these concerns, limited published literature is available quantifying radiation exposure associated with the various intraoperative limb length and femoral offset assessment techniques used during DAA-THA. Although fluoroscopy use is understandably influenced by surgeon experience, there remains a need to evaluate radiation exposure in a systematic and quantifiable manner. Therefore, the primary purpose of this study was to quantify fluoroscopy use between two commonly used intraoperative limb length and femoral offset assessment techniques during DAA-THA performed by a single orthopedic surgeon. The secondary purpose was to evaluate the role of body mass index (BMI) in radiation use during DAA-THA, as the radiation dose required by image intensifiers typically must increase to maintain image quality.

## Materials and methods

The study was approved by the Sanford Health Institutional Review Board (ID: STUDY00003278). A retrospective review was conducted of a series of adult patients (age >18 years at the time of surgery) who underwent DAA-THA performed by a single orthopedic surgeon between January 2016 and May 2023 at a single institution. Potential study subjects were identified using the current procedural terminology (CPT) code 27130 for THA. Inclusion criteria consisted of patients who underwent primary THA for osteoarthritis using a cementless DAA-THA technique, with available intraoperative fluoroscopic images and corresponding radiation exposure reports. Patients with orthopedic hardware in the operative hip, polytrauma, revision THA, hip hemiarthroplasty, cemented THA, or cases lacking intraoperative imaging with radiation exposure documentation were excluded.

All THAs were performed using a standard DAA on an orthopedic surgery table, with intraoperative assessment of implant position. Two intraoperative measurement strategies were employed to assess leg length and femoral offset, both of which were derived from the radiographic overlay technique described by Matta et al. [13]. In the first technique, the contralateral hip was used as the reference image. This method involved obtaining a baseline fluoroscopic image of the nonoperative hip, followed by adjustment of the operative hip to match the profile of the proximal femoral landmarks. In the second technique, the ipsilateral (operative) hip served as the reference, and a baseline fluoroscopic image was acquired after surgical exposure but before femoral neck osteotomy. Of note, when using the ipsilateral reference technique, the C-arm height and orientation were kept constant throughout the case after the initial imaging. In both methods, the captured fluoroscopic image was printed on X-ray film [13]. For the contralateral method, the acquired image was laterally inverted (mirrored) before printing on film so that it could subsequently be overlaid with the operative hip film. A permanent marker was used to trace pelvic landmarks on the pre-prosthetic film, including the iliopectineal line, inferior pubic ramus, inferior border of the ischial tuberosity, greater trochanter, and lesser trochanter. After fitting with trial implants, an anteroposterior image of the operative hip was printed and traced with a marker in a similar fashion. The pre-prosthesis and post-prosthesis films with the marked reference landmarks were then overlaid and placed on an X-ray illuminator, allowing the surgeon to compare congruency and determine whether any adjustments to the final implants were needed to achieve the desired leg length and femoral offset. A final pelvic radiograph was obtained intraoperatively for both techniques. The vertical height difference between the inferior rami and a selected point on the lesser trochanters was used to assess final leg length. The choice of technique was based on surgeon preference during each operative period. Aside from the assessment technique used, all other aspects of the surgical approach, implant selection, and perioperative protocols remained consistent across cases.

The retrospective chart review collected patient demographic information along with relevant intraoperative data. Intraoperative variables included fluoroscopy radiation dose, fluoroscopy radiation time, and total operative time. Fluoroscopy time and radiation dose for each case were obtained from the intraoperative C-arm exposure report documented in the patient’s medical record. Operative time, defined as the interval between the initial skin incision and final skin closure, was used as a surrogate indicator of case complexity.

Data analyses

Descriptive statistics, including the mean, standard deviation, and range, were calculated for all variables. Fluoroscopy time, radiation dose, and operative time were evaluated using an unpaired two-tailed t-test. Fluoroscopy and radiation data expressed as ratios normalized to operative time, which served as a surrogate for case difficulty, were also evaluated using an unpaired two-tailed t-test. Data stratified by BMI category were evaluated using a two-way analysis of variance (ANOVA) with technique and BMI as factors. Due to the limited sample size following stratification, post hoc multiple-comparison testing was not performed. The Pearson correlation coefficient (r) was used to examine the relationship between BMI and various intraoperative variables. Statistical significance was defined as an α level corresponding to P < 0.05. All data are expressed as mean ± standard deviation (SD), and analyses were performed using Prism 10.0 (GraphPad, Dotmatics, Boston, MA).

Patient demographics

A total of 264 THA cases were initially identified, of which 238 utilized the DAA. Patients who underwent cemented THA (29 cases), those lacking fluoroscopy documentation (34 cases), and those who underwent surgery for fracture or avascular necrosis (16 cases) were excluded. A total of 159 cases met the study inclusion criteria and were included in the final analysis. Of these cases, 77 patients (mean age, 63 years; mean BMI, 31.0 kg/m²; 52% men) comprised the contralateral reference technique group, and 82 patients (mean age, 64 years; mean BMI, 31.3 kg/m²; 51% men) comprised the ipsilateral reference technique group (Table 1). When evaluating BMI distribution, the contralateral group (n = 77) consisted of 1 underweight patient (BMI <18.5 kg/m²), 5 normal-weight patients (18.5 ≤ BMI <25.0 kg/m²), 34 overweight patients (25.0 ≤ BMI <30.0 kg/m²), 20 patients with class 1 obesity (30.0 ≤ BMI <35.0 kg/m²), 11 patients with class 2 obesity (35.0 ≤ BMI <40.0 kg/m²), and 6 patients with class 3 obesity (BMI ≥40.0 kg/m²). The ipsilateral method group (n = 82) included one underweight patient, eight normal-weight patients, 27 overweight patients, 26 patients with class 1 obesity, 14 patients with class 2 obesity, and 6 patients with class 3 obesity (Table 2).

**Table 1:** Patient demographics

	Contralateral technique (n = 77)	Ipsilateral technique (n = 82)
Sex		
Women	37 (48%)	40 (49%)
Men	40 (52%)	42 (51%)
Laterality		
Left	41 (53%)	38 (46%)
Right	36 (47%)	44 (54%)
Age (years; mean ± SD, range)	63 ± 8 (36-79)	64 ± 10 (34-84)
BMI (kg/m^2^; mean ± SD, range)	31.0 ± 5.3 (17.6-41.9)	31.3 ± 5.4(17.8-45.0)

**Table 2:** Patient demographics stratified by body mass index

BMI category	Variable	Contralateral technique	Ipsilateral technique
Underweight (BMI < 18.5 kg/m^2^)	Women (n)	1	-
	Men (n)	-	1
	Left (n)	1	1
	Right (n)	-	-
	Age (years)	62	61
	BMI (kg/m^2^)	17.64	17.78
Healthy (18.5 ≤ BMI <25.0 kg/m^2^)	Women (n)	5	4
	Men (n)	-	4
	Left (n)	1	6
	Right (n)	4	2
	Age (years) (mean ± SD)	62 ± 8	60 ± 13
	BMI (kg/m^2^) (mean ± SD)	22.5 ± 1.7	23.5 ± 1.5
Overweight (25.0 ≤ BMI <30.0 kg/m^2^)	Women (n)	15	16
	Men (n)	19	11
	Left (n)	17	11
	Right (n)	17	16
	Age (years) (mean ± SD)	64 ± 9	64 ± 10
	BMI (kg/m^2^) (mean ± SD)	27.8 ± 1.4	27.8 ± 1.5
Class 1 obesity (30.0 ≤ BMI <35.0 kg/m^2^)	Women (n)	8	11
	Men (n)	12	15
	Left (n)	12	13
	Right (n)	8	13
	Age (years) (mean ± SD)	62 ± 5	67 ± 9
	BMI (kg/m^2^) (mean ± SD)	32.4 ± 1.3	32.2 ± 1.4
Class-2 Obesity (35.0 ≤ BMI <40.0 kg/m^2^)	Women (n)	6	5
	Men (n)	5	9
	Left (n)	6	6
	Right (n)	5	8
	Age (years) (mean ± SD)	63 ± 7	60 ± 13
	BMI (kg/m^2^) (mean ± SD)	37.7 ± 1.2	36.7 ± 1.4
Class-3 Obesity (BMI ≥ 40.0 kg/m^2^)	Women (n)	2	4
	Men (n)	4	2
	Left (n)	4	1
	Right (n)	2	5
	Age (years) (mean ± SD)	59 ± 14	66 ± 5
	BMI (kg/m^2^) (mean ± SD)	41.1 ± 0.5	42.7 ± 1.6

## Results

The contralateral group had a mean fluoroscopy time of 60.4 ± 21.2 seconds (range, 28-139 seconds), a mean radiation dose of 15.4 ± 13.3 mGy (range, 3.4-85.2 mGy), and a mean operative time of 154.3 ± 28.3 minutes (range, 112-232 minutes). The ipsilateral group had a mean fluoroscopy time of 55.9 ± 19.6 seconds (range, 23-119 seconds), a mean radiation dose of 13.0 ± 9.0 mGy (range, 2.9-49.9 mGy), and a mean operative time of 164.6 ± 22.8 minutes (range, 119-220 minutes). No significant differences were observed between the groups in fluoroscopy time (P = 0.17; Figure 1a) or radiation dose (P = 0.17; Figure 1b). Operative time was significantly longer when the ipsilateral technique was employed (P = 0.01; Figure 1c). As a surrogate measure of case complexity, fluoroscopy time and radiation exposure for each case were normalized to operative duration by dividing each parameter by the corresponding total operative time. The contralateral group demonstrated a mean normalized fluoroscopy time of 0.39 ± 0.1 fluoroscopy time/operative time (arbitrary units, AU) and a mean normalized radiation dose of 0.10 ± 0.07 mGy/operative time (AU). The ipsilateral group demonstrated a mean normalized fluoroscopy time of 0.34 ± 0.1 fluoroscopy time/operative time (AU) and a mean normalized radiation dose of 0.08 ± 0.05 mGy/operative time (AU). Both normalized fluoroscopy time (P = 0.0025; Figure 1d) and normalized radiation dose (P = 0.045; Figure 1e) were significantly lower when the ipsilateral technique was used.

**Figure 1:**
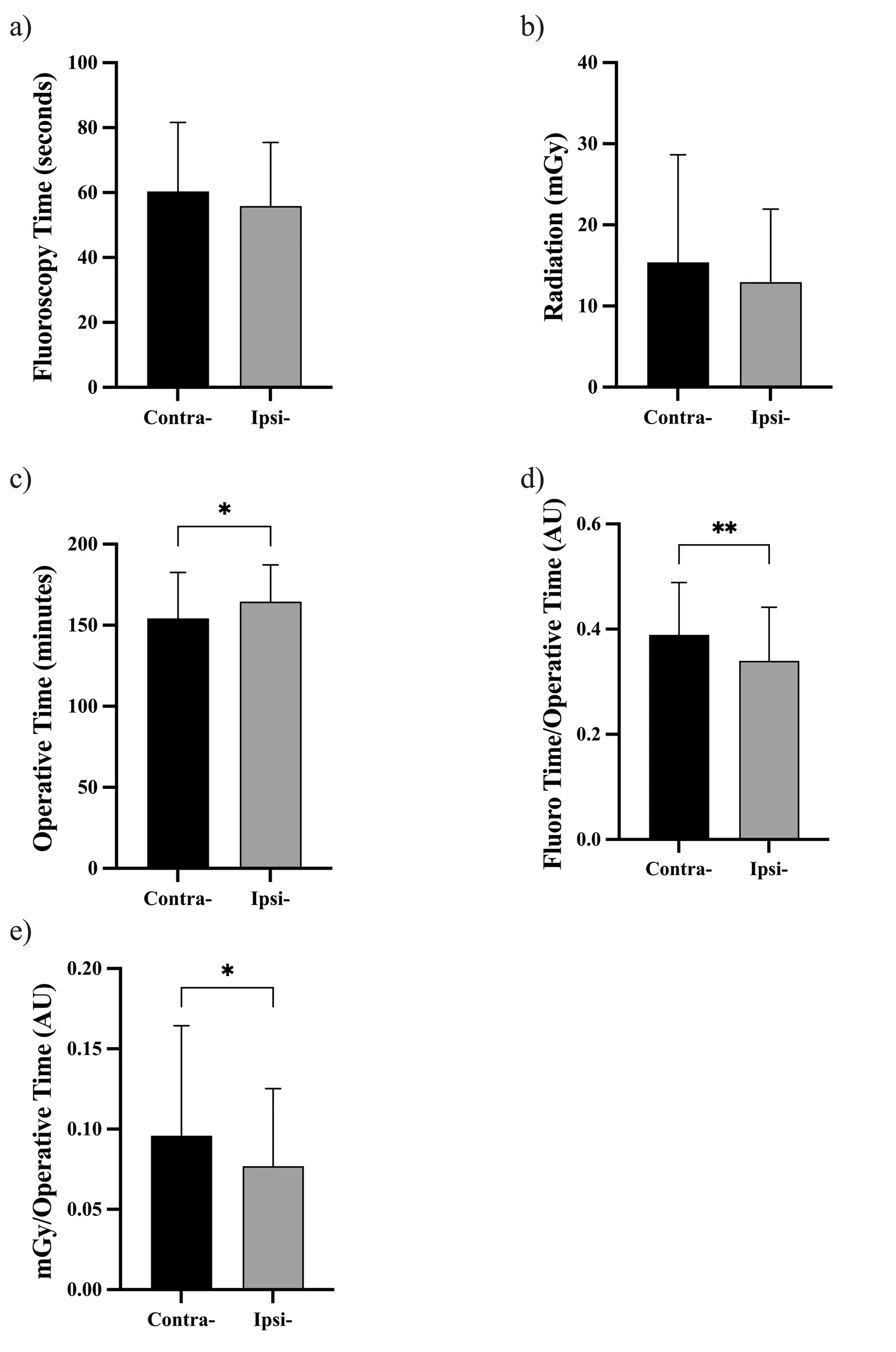
Intraoperative variables (a) Fluoroscopy time (seconds). (b) Radiation dose (mGy). (c) Operative time (minutes). (d) Normalized fluoroscopy time (AU, arbitrary units). (e) Normalized radiation dose (AU). As a surrogate measure of case complexity, fluoroscopy time and radiation exposure for each case were normalized to operative duration by dividing each parameter by the corresponding total operative time and are presented as arbitrary units (AU). Bars represent statistical significance between the contralateral and ipsilateral techniques. P < 0.05. Data are expressed as mean ± standard deviation.

When fluoroscopy time was stratified by BMI category, no significant main effect of BMI or technique, or interaction between the two variables, was observed (P > 0.05; Figure 2a). In contrast, radiation dose stratified by BMI demonstrated significant main effects of BMI (P < 0.001) and technique (P = 0.001) (Figure 2b). Additionally, radiation dose demonstrated a significant interaction between BMI and technique (P = 0.03; Figure 2b). When operative time was stratified by BMI, significant main effects were observed for both BMI (P < 0.001) and technique (P = 0.04), but no significant interaction between the variables was observed (P > 0.05) (Figure 2c). Two patients, one from each technique group, were excluded from the BMI-stratified analysis because each was the sole representative of the underweight category. Additionally, only main effects and interactions were assessed following the two-way ANOVA because of the limited sample size after stratification. The relationship between fluoroscopy time and BMI demonstrated a significant but weak correlation (r = 0.18, P = 0.02, 95% CI, 0.02809-0.3292; Figure 3a). Radiation dose (r = 0.52, P < 0.0001, 95% CI, 0.3939-0.6232; Figure 3b) and operative time (r = 0.42, P < 0.001, 95% CI, 0.2858-0.5426; Figure 3c) both demonstrated significant, moderate correlations with BMI.

**Figure 2:**
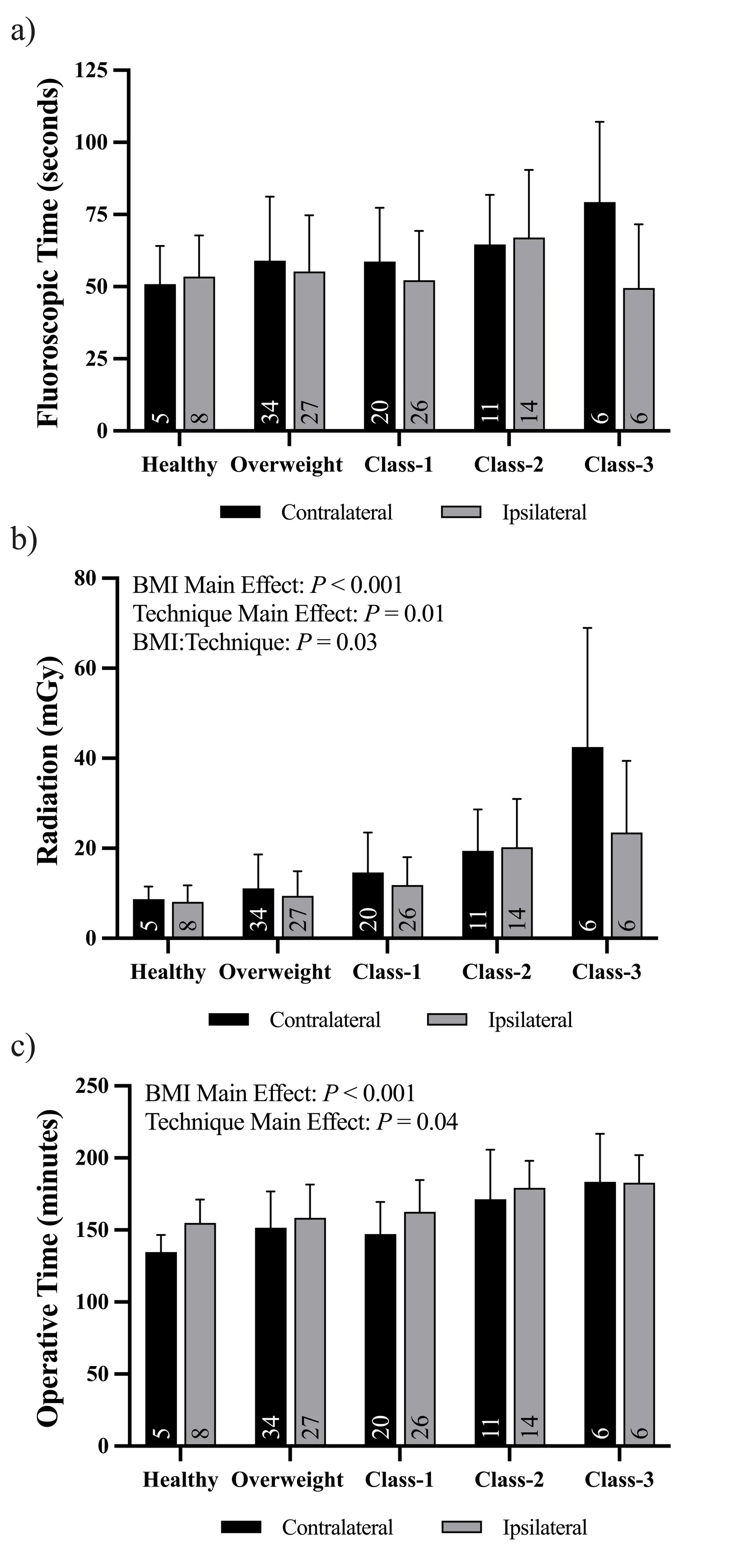
Intraoperative variables stratified by body mass index (BMI) (a) Fluoroscopy time (seconds). (b) Radiation dose (mGy). (c) Operative time (minutes). Main effects and their associated P-values are presented for BMI and/or technique within the respective dataset when statistically significant. Significant interactions between BMI and technique are indicated with their respective P-values. Data are expressed as mean ± standard deviation. The value within each column represents the corresponding sample size following stratification by BMI.

**Figure 3:**
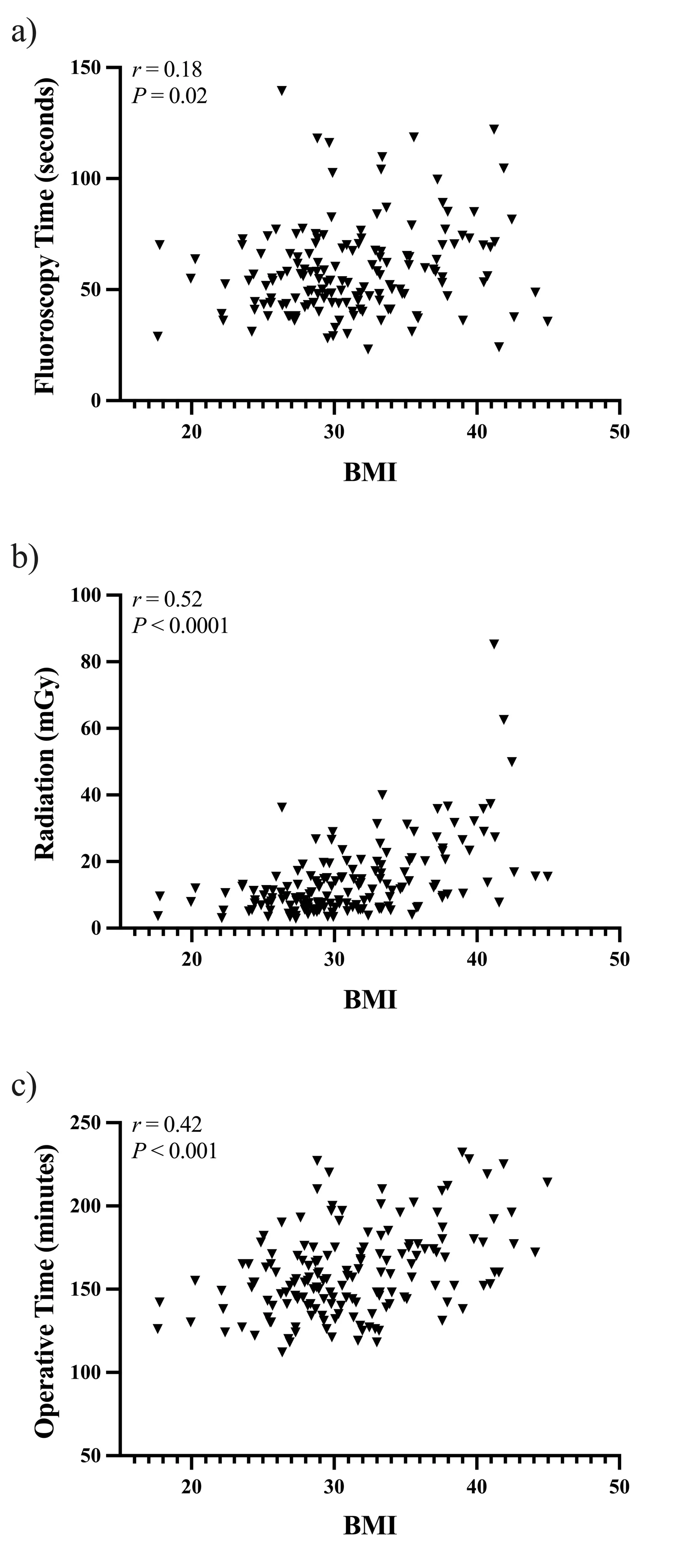
Correlation with body mass index (BMI) (a) Fluoroscopy time (seconds). (b) Radiation dose (mGy). (c) Operative time (minutes). Pearson’s correlation coefficient (r) and the associated P-value are presented within the respective dataset.

## Discussion

The present study is, to our knowledge, the first to report intraoperative fluoroscopy time, radiation exposure, and operative time between two limb-length assessment techniques during DAA-THA performed by a single surgeon. Additionally, this study complements the existing literature regarding the role of BMI in increased intraoperative fluoroscopy use.

In the present dataset, both the contralateral and ipsilateral reference techniques, when performed by a single surgeon, yielded comparable fluoroscopy times and radiation exposure. The use of the contralateral hip as a radiographic control for assessing operative limb length and femoral offset was first described in detail by Matta et al. [13]. In contrast to using the operative hip as a reference, one could argue that more fluoroscopic images may be obtained during repositioning of the operative limb when the contralateral hip is used as the reference. A modified version of this radiographic overlay technique was later described in which the operative hip is used as the reference, particularly in the setting of advanced degenerative changes or prior surgical intervention involving the contralateral hip, which could affect the interpretation of native hip anatomy [14,15]. Ultimately, our single-surgeon dataset indicates that both techniques can be used efficiently without compromising radiation safety.

Operative time was significantly longer with the ipsilateral technique. However, this difference is unlikely to be clinically significant when accounting for the broad variability inherent in surgical procedures. Interestingly, after controlling for operative time, the ipsilateral technique was associated with significant reductions in both fluoroscopy duration and overall radiation dose. Although using operative time as a proxy for case complexity has clear limitations, controlling for operative time helps isolate radiation utilization from the effects of prolonged procedure duration. Nonetheless, because the absolute reductions were modest and operative duration is subject to inherent stochastic factors, such as surgeon experience, patient BMI, operating room efficiency, and trainee participation, the clinical impact of this reduction remains uncertain. Ultimately, given our nonrandomized, retrospective study design, these hypothesis-generating findings should be interpreted with caution and highlight the need for further studies to clarify the relationship between surgical complexity and radiation utilization during DAA-THA.

Higher BMI has consistently been associated with significantly greater radiation dose during fluoroscopically guided procedures [16-19]. Image intensifiers automatically increase exposure to maintain image quality in patients with higher BMI, and thus, radiation dose can increase with BMI even when fluoroscopy time does not change [18,19]. In the present study, following stratification by BMI category, we observed no significant main effect of BMI or technique on fluoroscopy time. Regardless of technique, only a very weak correlation was observed between BMI and fluoroscopy time. However, significant main effects of both BMI and technique were observed for radiation dose and operative time. Unfortunately, the sample size in our dataset was limited following stratification by BMI category, and therefore, no reliable post hoc comparisons could be performed to further explore the significant interaction between BMI and technique on radiation dose. In contrast to fluoroscopy time, BMI demonstrated a significant positive relationship with both radiation dose and operative time. These findings appear to be consistent with the existing literature, in which BMI appears to affect radiation dose without strongly influencing fluoroscopy time [18-20], as well as with prior orthopedic literature highlighting the increased operative time required in patients with higher BMI [21-23]. Despite these findings, data regarding intraoperative fluoroscopy stratified by BMI category during DAA-THA remain sparse, with mixed conclusions among the available literature [24]. A single study of 157 patients demonstrated a positive correlation between BMI and radiation dose [25]. In contrast, a smaller THA study did not report a significant correlation between BMI and radiation dose [26,27]. Outside of THA, patients with higher BMI have required increased radiation doses during periacetabular osteotomy and arthroscopic hip preservation procedures [28-30]. Additionally, empirical data suggest greater radiation exposure to staff during hip procedures in patients with obesity, further supporting the need to explore this relationship [27]. The findings of our study demonstrate the complex interplay between patient habitus, procedural duration, and radiation exposure, highlighting the need for larger prospective cohorts to fully delineate how technical adjustments can minimize radiation exposure in patients with higher BMI.

Although numerous techniques exist for the intraoperative assessment of limb length and femoral offset, empirical data regarding the resultant radiation exposure to patients and surgical personnel remain limited. The acute radiation exposure associated with DAA-THA is generally considered low, approximately one-fourth that of a standard chest CT [25]. However, this should not diminish efforts to minimize radiation exposure, particularly given the cumulative lifetime dose incurred by patients, surgeons, and operating room staff. While distance from the radiation source and personal protective equipment (e.g., lead aprons) can attenuate exposure by up to 99%, orthopedic surgeons who routinely use fluoroscopy face a fivefold increase in lifetime cancer incidence [12], with unshielded body areas such as the hands and eyes bearing the greatest risk [10,11]. The Centers for Disease Control and Prevention (CDC) advocates maintaining radiation exposure As Low As Reasonably Achievable (ALARA). Adhering to these principles requires ongoing evaluation of techniques that reduce intraoperative radiation exposure without compromising surgical accuracy or patient safety. Although our study did not directly measure occupational radiation dose to operating room personnel, minimizing overall fluoroscopy parameters inherently reduces the occupational hazards associated with intraoperative imaging. This represents an area that warrants continued investigation.

This study is not without limitations. First, its retrospective, nonrandomized design and evaluation of a single surgeon’s series may limit the generalizability of our findings. Second, we acknowledge the potential for learning-curve bias, as the ipsilateral technique was predominantly utilized during the latter half of the study period and may therefore partially reflect overall procedural proficiency. Third, image intensifier settings were not retrospectively standardized; to account for this variability, total fluoroscopy time was analyzed alongside radiation dose. Fourth, using operative time as a surrogate for case complexity represents an imperfect proxy, as operative duration may be influenced by confounding factors, including evolving surgeon proficiency with the procedure, operating room efficiency, trainee participation, and patient characteristics such as BMI. Fifth, although not the primary objective of this investigation, final clinical and radiographic outcomes, such as limb-length discrepancy and restoration of femoral offset, were not assessed between the two techniques. Finally, the limited sample size following stratification by BMI category reduced statistical power for certain post hoc comparisons.

## Conclusions

Although prior studies have evaluated fluoroscopy use during DAA-THA, direct comparison of these two reference techniques is unique to this study. Both reference techniques demonstrated similar fluoroscopy use, while the contralateral technique was associated with shorter operative time. However, after controlling for case duration, the ipsilateral technique demonstrated lower overall fluoroscopy use, although further investigation is warranted. Across all cohorts, elevated BMI consistently increased radiation exposure and operative duration. Future work should focus on larger, prospective cohorts to assess the interplay between case complexity, radiographic accuracy, and real-time occupational exposure to operating room staff. Ultimately, ongoing critical evaluation of fluoroscopic workflows remains imperative to lowering radiation exposure to patients and surgical teams without compromising surgical safety.
